# Effects of Depressed Mood on Syllogistic Reasoning: The Buffering Role of High Working Memory Span

**DOI:** 10.3389/fpsyg.2021.645751

**Published:** 2021-09-27

**Authors:** Jaroslaw Wasielewski, Klara Rydzewska, Grzegorz Sedek

**Affiliations:** Interdisciplinary Center for Applied Cognitive Studies (ICACS), Institute of Psychology, SWPS University of Social Sciences and Humanities, Warsaw, Poland

**Keywords:** mental models, syllogistic reasoning, conflict and no-conflict syllogisms, working memory capacity (WMC), depressed mood

## Abstract

Previous research provided consistent evidence for the existence of the unique cognitive limitation in depressed mood: the impairment of the construction of mental models. In the current research, we applied the classical paradigm using categorical syllogisms to examine the relationship between depressed mood and integrative reasoning, aiming at gathering research evidence on the moderating role of the operation span of working memory. Specifically, we examine the hypothesis that high working memory capacity is a buffering variable and acts as a protective factor preventing the negative impact of depressed mood on syllogistic reasoning. A categorical syllogism, in the simpler evaluative form, consists of two premises (that are assumed to be true) and a conclusion that is to be evaluated as valid (when it follows logically from the premises) or invalid (when it does not follow from the premises). In the cover story, we informed participants that they would read about some observations carried out in a normal garden (believable conclusions) versus in a garden with radical genetic transformations (unbelievable conclusions) in order to stimulate the emergence of belief bias. The participants were 115 high school students who filled out the BDI scale and completed the OSPAN task. In line with predictions, there were main effects of depressed mood and operation span on the accuracy of performance (worse performance in the group with a high in comparison to a low level of depressed mood and much worse performance in low compared to high OSPAN participants). The analyses yielded a strong interaction effect of Depressed mood × OSPAN × Conflict. For participants with high levels of working memory capacity, there were no limitations related to a high level of depressed mood in syllogistic reasoning. On the other hand, a different pattern emerged for participants with low working memory span. In this group, participants with a high level of depressed mood in comparison to those with a low level of depressed mood showed much higher limitations in syllogistic reasoning, especially in reasoning concerning conflict syllogisms. We discuss the implications of this research for recent therapeutic programs using computerized cognitive tasks aimed at individuals with a high level of depressed mood.

## Introduction

Numerous recent research has indicated multiple ways in which emotions affect the quality of higher order cognitive processes such as reasoning (for reviews, see Blanchette and Richards, 2010; Blanchette, 2014). Most of the classical and recent studies demonstrated that emotions not linked semantically to the content of reasoning tasks impair the accuracy of deductive reasoning (Channon and Baker, 1994; Blanchette and Richards, 2004; Blanchette and Leese, 2011; Eliades et al., 2012; Jung et al., 2014; Trémolière et al., 2016; Blanchette and Nougarou, 2017; Caparos and Blanchette, 2017; Viau-Quesnel et al., 2019). Recent and replicated research also showed the original evidence that in conditions where emotional contents are personally relevant emotions often improve logical reasoning (Johnson-Laird et al., 2006; Goel and Vartanian, 2011; Blanchette and Campbell, 2012; Blanchette and Caparos, 2013; Blanchette et al., 2014).

In this paper we examine the impact of depressed mood^1^ on categorical syllogisms rich in the content, but not related semantically to depressive thoughts or symptoms. The present research aims at gathering further empirical evidence for the main prediction of the cognitive exhaustion model (von Hecker and Sedek, 1999; Sedek and von Hecker, 2004; von Hecker et al., 2013) that depressed mood impairs generative processes of building mental models. We argue that a high level of depressed mood affects not only deductive reasoning processes (often explained by mental models’ theoretical accounts), but also mental models of social relations which are not reasoning processes per se but rather applications of social rules of sentiment relations (c.f., von Hecker and Sedek, 1999). The notion of *depression* is frequently employed to describe a broader category of depressive symptoms, depressed mood, dysphoria, and the depression syndrome as such (Joormann, 2005). Numerous debates in the literature have addressed the question of whether minor depression symptoms (like depressed mood assessed by the BDI) differ quantitatively or qualitatively from severe clinical depression and the available data are generally consistent with the hypothesis of continuity (Flett et al., 1997; de Graaf et al., 2010; Lee et al., 2013). Depression is characterized by persistent negative mood and specific deficits in cognitive functioning (Joormann and Arditte, 2014; Rock et al., 2014; Trivedi and Greer, 2014). These deficits include “ruminative” thinking, recurring ideas and thoughts with negative or self-devaluing content. Moreover, such deficits involve individuals with depression experiencing limitations in solving complex cognitive problems. There are a number of explanations for an impaired performance of complex tasks in depressed individuals; drawing on cognitive resources or memory limitations (Hasher and Zacks, 1979; Weingartner, 1986; Burt et al., 1995; Gotlib et al., 1996), impaired inhibition (Hertel, 2004; Joormann, 2005), lowered efficiency of cognitive strategies (Hartlage et al., 1993; Smith et al., 1993; Kofta and Sedek, 1998; von Hecker and Sedek, 1999), or lack of cognitive initiative (Hertel and Hardin, 1990; Hertel, 1997) as explanatory concepts.

In the first part of this introduction, we overview the assumptions of the cognitive exhaustion model and summarize gathered evidence of limitations related to a high level of depressed mood in building mental models. Next, we formulate specific predictions based on this cognitive exhaustion model that depressed mood impairs correct solving of conflict syllogisms but not no-conflict syllogisms (e.g., De Neys and Van Gelder, 2009) and that this relationship is moderated by the operation span of working memory (OSPAN, Turner and Engle, 1989).

The cognitive exhaustion model (Sedek and Kofta, 1990; Sedek et al., 1993; Kofta and Sedek, 1998) assumes that people are likely to engage in systematic mental activity when dealing with problem-solving situations. They attempt to understand the meaning of task demands, they notice and pay attention to diagnostic pieces of information, detect regularities or inconsistencies, formulate and examine hypotheses, and so forth. However, in uncontrollable surroundings, such activity remains futile because it cannot lead to real progress in problem solving. By definition, in unsolvable situations, no reliable explanatory rules can be found for solving problems. Therefore, although individuals might generate quite a few preliminary hypotheses, they would eventually not be able to differentiate between good and poor ideas in seeking a solution. It is hypothesized (see: Sedek and Kofta, 1990; Kofta and Sedek, 1998; von Hecker and Sedek, 1999; von Hecker et al., 2013) that prolonged cognitive effort without “cognitive gain” results in an altered psychological state, which we term cognitive exhaustion. The essential quality of this transitory state is a generalized impairment of constructive and integrative mental processing. Therefore, after uncontrollable pre-exposure subjects’ ability to form new ideas and generate hypotheses is diminished. In terms of general adaptive functions, cognitive exhaustion states seem especially disruptive to more complex problem solutions requiring non-routine, flexible steps of processing in either achievement or interpersonal domains. It is important to note that a number of researchers found close parallels between some aspects of cognitive functioning in depression and the state resulting from pre-exposure to uncontrollability (Seligman, 1975; Abramson et al., 1978; Kuhl, 1984; Pittman and D’Agostino, 1989; Flett et al., 1997). In line with our own cognitive exhaustion model (Kofta and Sedek, 1998), we assume that some of the cognitive impairments observed in depression can be explained in terms of experienced uncontrollability. This experience may stem from past, irreversible life events, from subsequent ruminating, or from counterfactual thinking (Niedenthal et al., 1994; Davis et al., 1995). It is hypothesized that uncontrollability and, in particular, ruminative thoughts about uncontrollable conditions, lead to a depletion of those cognitive resources that support generative and flexible, constructive thinking. Constructive thinking may still be initiated by depressive individuals, at times even more vigorously than by non-depressive individuals. Nevertheless, it might yield less success in terms of the quality of new, integrative constructions, such as mental models.

In line with the classic idea of Johnson-Laird (1983), a mental model is defined here as a construction based on incoming data (such as premises in a reasoning task, or text sentences in comprehension tasks). Mental models are generated on-line during task performance. This constructive activity might be contrasted to mere preservation of presented information. Mental models are episodic representations, resembling structural and functional relations of real entities (Brewer, 1987). They may function in support of analog simulations of the events in the world, either real or imaginary (Greeno, 1989). The existing research evidence for generative reasoning problems among depressed individuals comes from our studies on the construction of mental models. In the first studies (von Hecker and Sedek, 1999), we employed a process tracing method in order to study the construction of social mental models in depressed and nondepressed participants. We focused on mental models involving a set of perceived sentiment relations. There is experimental evidence (Hummert et al., 1990; von Hecker, 1997) showing that sets of sentiment relations, such as, “Andrew and Bert like each other,” “Andrew and Chris dislike each other,” “Chris and Danny like each other,” are simultaneously represented in memory by means of the so-called mental cliques. In this case, the correctly constructed mental model should consist of two cliques: Andrew + Bert in the first clique and Chris + Danny in the second clique. Members of each clique like each other and dislike members of the other clique. These structures are constructed from single relations, in a process guided by a step-by-step integration of more or less diagnostic information. The results of this research (von Hecker and Sedek, 1999) showed that the quality of the constructed social mental models was impaired by depression. More specifically, depressed participants (in comparison to nondepressed participants) made more incorrect inferences in terms of the actual number of cliques, and their allocation of individual target persons to particular cliques was less accurate. The findings for study times showed that depressed participants, like nondepressed participants, allocated more study time to diagnostic relations than to nondiagnostic relations, thereby recognizing the diagnostic value of some specific types of relations. However, unlike nondepressed participants, depressed participants did not use diagnostic relations adequately for the purpose of model construction.

The next series of studies (Sedek and von Hecker, 2004; Brzezicka et al., 2017) using linear syllogisms provided a conceptual replication and an extension of these findings. In this experimental procedure participants were asked to study three pairs of relations in each trial, for example, “John is taller than David,” “David is taller than Brian,” and “Brian is taller than Robert.” An integrated mental model representation of such a set of pairs would always be a linear order “John > David > Brian > Robert” (John is the tallest, Robert is the shortest). Immediately after the presentation of the three pairs, participants were tested on all possible pairs within the order, for example, Brian—Robert (adjacent pairs, which had been learned), John—Brian (two-step relations), and Robert—John (endpoint relations), by prompting participants with statements in either a false (e.g., “Robert is taller than John”) or correct format (e.g., “Brian is taller than Robert”), and asking them for quick verification. The difficulty of integrating the three pairs was varied by administering sequences in which subsequent pairs always had an element in common by which the two could be linked (e.g., “David is taller than Brian” being presented after “John is taller than David”), versus other sequences in which the pairs were presented in a scrambled way such that there was less overlap of elements between subsequent pairs.

The results of these studies were clear (Sedek and von Hecker, 2004, subclinical depression; Brzezicka et al., 2017, clinical depression). For the nondepressed group, there was a constant high level of accuracy across analyzed pair distances (adjacent, two steps, endpoint). It strongly suggests that in this group, participants tended to retrieve their answers from an integrated model, as queries on inferred endpoint relations were answered with no less accuracy than explicitly learned, adjacent ones. On the other hand, in the depressed group, there was a substantial decrease in accuracy from explicitly learned to inferred relations. Given this pattern, we concluded that depressed individuals did not spontaneously integrate the pairs during learning, but retrieved the pairs at the time of the query to make transitive inferences at this later point in time. It is of further interest to note that despite the apparent differences in terms of achieved mental model construction, both groups showed strikingly similar behavior during learning. Namely, overall study times were similar in both groups. As further analyses showed, it took both groups longer to study pairs from more difficult orders than pairs from easier orders, and for both groups, this was particularly the case when studying any third pair in the sequence. The observation that both groups exerted similar amounts of time in solving the linear syllogisms tasks is consistent with previously discussed findings on social cliques models (von Hecker and Sedek, 1999).

Categorical syllogisms have been widely studied since the beginning of experimental psychology as the prototype of logical reasoning tasks (Woodworth and Sells, 1935; Begg and Denny, 1969; Gilhooly et al., 1993). Using the example from the study of Gilhooly et al. (1993), a typical syllogistic reasoning problem involved two statements (premises) that are assumed to be true, such as “All dogs are mammals” and “All corgis are dogs,” and the participants’ task is to decide what conclusion can be reached based on those premises. In this case, the valid conclusion is “All corgis are mammals.” This version of the syllogism task where the conclusion has to be constructed is very difficult for participants (Gilinsky and Judd, 1994). More frequently used versions of syllogism tasks are the ones that demand to evaluate only whether the presented conclusion is valid or invalid. According to the classical theory of mental models (Johnson-Laird, 1983), participants in the beginning construct a mental model in which the premises are perceived as true and then formulate a tentative conclusion consistent with the model that relates terms not presented in the premises. In the next step, participants search for counterexamples in which the initial conclusion is false, and if so, they continue to search for an alternative mental model. According to Johnson-Laird (1983), the difficulty of drawing a valid conclusion depends on the number of mental models required for a thorough search of a final conclusion. The larger the number of tentative mental models, the greater the difficulty of a given syllogism and the greater strain on working memory. The working memory system enables the simultaneous representation and manipulation of information, hence replicated findings demonstrated strong correlations between different measures of working memory capacity and syllogistic reasoning and those correlations were stronger for multiple model syllogisms (e.g., Gilhooly et al., 1993; Gilinsky and Judd, 1994; Quayle and Ball, 2000; Copeland and Radvansky, 2004; Handley et al., 2004).

Such established findings inspire to examine the potential role of working memory limitation as the explanation of impaired performance in syllogism tasks among participants with a high level of depressed mood. That is, although participants with a high level of depressed mood may attempt to combine the premises of syllogism tasks, the effective integration into a logical conclusion may be expected to exceed the capacity of their (but not participants’ with a low level of depressed mood) working memory. The previous research on depression and linear syllogisms (Sedek and von Hecker, 2004) showed that depression (measured by the Beck Depression Inventory, BDI, Beck, 1967) is not correlated with the OSPAN. Therefore, we assume it is rather improbable that working memory mediates the relationship between depressed mood and limitations in syllogistic reasoning. However, the role of working memory as the moderator of the influence of depressed mood on impaired syllogistic reasoning is more plausible. Namely participants with a relatively high operation span (both with high and low levels of depressed mood) might solve categorical syllogisms pretty well and on a similar level. However, among participants with relatively low working memory capacity, reasoning accuracy might be significantly lowered in participants with a high level of depressed mood compared to those with a low level of depressed mood. To examine this prediction of working memory as the moderator of the relationship between depressed mood and limitations in syllogistic reasoning, the syllogism problems in this study consist of both one-model syllogisms and three-model syllogisms, and the differences in their performance are examined for their relations (main effects and interaction effect) with both depressed mood (as measured by the BDI) and operation span of working memory (OSPAN).

Another approach to an explanation of limitations related to a high level of depressed mood in syllogistic reasoning underlines the content effects causing belief bias in reasoning that might especially affect participants with a high level of depressed mood. The replicated series of studies demonstrated that participants’ personal beliefs of knowledge of the world impaired their ability to reason logically in syllogistic tasks. That is, when presented with conclusions to evaluate, participants were inclined to accept conclusions they believed to be true despite the clear instructions to rely on the logical reasoning only (classical references: Morgan and Morton, 1944; Oakhill and Johnson-Laird, 1985; Evans, 1989; Gilinsky and Judd, 1994). To put it in another way, participants’ problem is that belief-based reasoning often prompts incorrect responses that are in conflict with the logically appropriate response. For example, consider the following syllogism (from Gilinsky and Judd, 1994): “If all mothers are female and all parents are mothers, then all parents are female.” This conclusion is logically valid but unbelievable. According to the standard logic, this conclusion should be accepted as correct, however, many participants tend to reject it because it is unbelievable. Correct reasoning demands that this prepotent belief-based response is inhibited. Another example of belief-logic conflict is when participants should assess believable conclusions as logically invalid as in the following syllogism (again from Gilinsky and Judd, 1994): “If all sour fruits are citrus fruits and all citrus fruits are lemons, then all lemons are sour fruits.”

We have serious objections about the construction of syllogisms in such a way that they contain obviously *false* assumptions concerning one of the premises (in the previous examples, the premise “all parents are mothers” or “all sour fruits are citrus fruits” are obviously *false*). According to our concerns, presenting participants with premises that are false in light of the objective reality might diminish their motivation to participate in research and therefore limit their involvement in effortful thinking. Therefore, in the cover story of our syllogism task, we informed participants that they would read about some observations carried out in a normal garden (believable conclusions) versus in a garden with radical genetic transformations (unbelievable conclusions) in order to stimulate the emergence of belief bias. The observations (premises) themselves were to be taken as valid; however, the participants’ task was to decide whether the conclusions following from them, made by the gardener, were logically correct (valid) or incorrect (invalid). Consider the exemplary unbelievable and valid syllogism from our study (see Table 1): “If all grapes are ripe and everything ripe glows in the dark, then all grapes glow in the dark.” The conclusion that “all grapes glow in the dark” and premise “everything ripe glows in the dark” are unbelievable but are probable (not obviously false) in the garden with radical genetic transformations.

**TABLE 1:** Examples of believable syllogisms (normal garden) and unbelievable syllogisms (garden with genetic transformations), valid vs. invalid, and relatively simple (one mental model) vs. relatively complex (three mental models).

Validity	Abstract form	Believable (normal garden)	Unbelievable (garden with genetic transformations)
Valid 1MM	All A are B All B are C →All A are C	All grasses are green Everything green is alive → All grasses are alive	All grapes are ripe Everything ripe glows in the dark → All grapes glow in the dark
Invalid 1MM	All A are B All B are C →All C are A	All the wormy fruits fall from the tree All the fruits that fall from the tree are overripe → All overripe fruits are wormy	All ants have wings Everything winged sings → Everything that sings is an ant
Valid 3MM	No A are B Some C are B → Some C are not A	No dogs have colorful feathers Some ducks have colorful feathers → Some ducks are not dogs	No edible product is a toadstool Some jumping mushrooms are toadstools → Some jumping mushrooms are not edible
Invalid 3MM	No A are B Some C are B → Some A are not C	No hare is a fox Some devious animals are foxes → Some hares are not devious	No smiling apples grow on the bushes Some pretty fruits grow on bushes → Some smiling apples are not pretty fruits

*For illustration purposes, the abstract form of such syllogisms is also presented. Conflict syllogisms are indicated in italics, no-conflict syllogisms have normal fonts.*

In our research half of the problems, referred to as conflict syllogisms, conflicted with believability as in the above example. For the other half of the problems, referred to as no-conflict syllogisms, the logical status of the conclusion was consistent with its believability. The inclusion of the no-conflict syllogisms allows for the direct examination of the role of efficiency of inhibition processes in correct reasoning. Consider the following example (again, from our set of syllogisms, see Table 1): “If all grasses are green and everything green is alive, then all grasses are alive.” The logical structure of this syllogism is the same as in the previous example; however, in this case, beliefs and logic are not in conflict. Hence, the correct response can be based on intuitive thinking without any need to inhibit the belief-based system.

Channon and Baker (1994) (who similarly to our study investigated participants with relatively low and relatively high scores on BDI) demonstrated that depressed participants showed significantly lower performance when solving abstract syllogisms compared to nondepressed participants. However, in their research, they do not refer to belief bias effects and do not analyze the role of individual differences in working memory. Interestingly, they suggested that these depression-related limitations might be related to the working memory limitations of depressed participants but did not demonstrate any direct research evidence for such a prediction. The goal of our research is to relate the issue of limitations related to a high level of depressed mood in solving classical syllogisms to the newer research domain of the role of distinction of no-conflict and conflict syllogisms and examining the role of working memory span. To date, according to our knowledge, there has been no research that combines depressed mood and working memory span as predictors of the no-conflict and conflict syllogisms. Because conflict syllogisms are cognitively more complex than no-conflict syllogisms, in line with the predictions of the cognitive exhaustion model we predict that a high level of depressed mood should impair syllogistic reasoning of conflict syllogisms but should not interfere with solving no-conflict syllogisms. Extending our previous prediction that among participants with relatively low working memory the reasoning accuracy might be significantly lowered among participants with a high level of depressed mood in comparison to participants with a low level of depressed mood, we predict that it should be demonstrated especially for conflict syllogisms (while solving no-conflict syllogisms should not be impaired). However, for participants with relatively high working memory, a high level of depressed mood should not interfere with the accuracy of solving even conflict syllogisms. These predictions are supported by the findings that measures of working memory capacity (and especially different versions of span tasks of working memory, such as Counting Span, Reading Span, or OSPAN) are strong predictors of the quality of syllogistic reasoning (Gilhooly et al., 1993; Gilinsky and Judd, 1994; Quayle and Ball, 2000; Copeland and Radvansky, 2004; Handley et al., 2004; Ding et al., 2020). To succinctly summarize our main predictions, we hypothesize that the relationship between depressed mood and syllogistic reasoning will be moderated by working memory capacity and format of syllogisms (conflict versus no-conflict).

To address motivational issues, participants with a high versus low level of depressed mood were examined in terms of time spent on solving syllogisms. Similarly to the previous research on the effect of subclinical depression on solving linear syllogisms (Sedek and von Hecker, 2004), we expected very similar patterns for participants with high and low levels of depressed mood. Hence, we assume comparable motivation to tasks in participants with high and low levels of depressed mood. Duration of processing time of both participants with low and high levels of depressed mood should be at comparable levels concerning the more extensive processing of more difficult syllogisms (M3) than simpler ones (M1) and, likewise, a longer studying of a second premise than the first premise (extra time for integrating premises), and especially the longest time for deciding whether conclusions are logically true (valid) or false (invalid).

## Methods

### Participants

The participants were 115 students from technical high school classes in Nowy Tomysl, a small town in western Poland (50 women, 65 men; mean age = 18.33, standard deviation = 0.47; 11 or 12 classes of formal education). Students were administered the Polish version of the Beck Depression Inventory (BDI; Beck, 1967). To facilitate interpretation of findings this sample was divided into two groups: low and high level of depressed mood, using a median split of BDI scores. The mean BDI scores for participants with a low level of depressed mood (*n* = 57) and a high level of depressed mood (*n* = 58) were 7.14 (SD = 3.18) and 22.53 (SD = 6.69), respectively^2^. The group difference on BDI scores was highly significant, Oneway *F*(1, 113) = 246.95, *p* < 0.001, *η_*p*_^2^* = 0.686. Mean BDI scores in the classical study of depressed mood and solving abstract syllogisms (Channon and Baker, 1994) were *M* = 2.5 for the nondepressed group and *M* = 19.1 for the depressed group. Hence, the means for the depressed mood group in the research of Channon and Baker (1994) were very similar to the mean scores in the current study.

## Materials

### Operation Span Task

Operation span task is a computerized measure of working memory capacity, which captures simultaneous maintenance and processing (Turner and Engle, 1989). Participants are presented with a series of arithmetic problems such as “Does (6 × 10) + 10 = 80?” followed by a word (e.g., “pencil”). The task is to correctly answer each problem and retain the word for later recall. Sequence length is increased from two to six. Our version of the task includes five sets of trials with three sequences each. Working memory capacity is calculated as the sum of correctly recalled words only in the sequences where all words were recalled correctly. Similarly as for the BDI scores, to facilitate interpretation of the findings this sample was divided into two groups using the median split of OSPAN on low OSPAN and high OSPAN participants^3^. The mean OSPAN scores for low OSPAN (*n* = 59) and high OSPAN (*n* = 56) participants were 21.32 (SD = 6.67) and 46.36 (SD = 8.07), respectively. The group difference on OSPAN scores was highly significant, Oneway *F*(1, 113) = 330.12, *p* < 0.001, *η_*p*_^2^* = 0.745.

### Computerized Syllogism Task

In the beginning of the computerized syllogism task the participants read the following instruction:

“Imagine you are a biologist. You just visited two gardens:

1. Owned by the Ecologists—in this garden it is made sure that everything is in harmony with nature.
2. Belonging to the Department of Genetics—here, in turn, scientists are working on new varieties of different types of plants and animals.

As an authority in the field of biology, you have been asked to express your opinion regarding the conclusions of the research taking place in both gardens. Two sentences will be displayed on the screen, one after another, followed by a conclusion. Decide whether this conclusion follows logically from the two previous sentences or not. To avoid being influenced by your own beliefs, the data for both gardens have been mixed up—so sometimes your observations will be consistent and sometimes not consistent with your daily experience.

Remember! Focus on whether or not the conclusions are correct, that is if they follow logically from the information provided in the premises.

DON’T GET SUGGESTED BY THE COMPLIANCE OF THE SITUATION WITH COMMON KNOWLEDGE, BUT ONLY WITH ITS LOGICAL CORRECTNESS.”

The participants received two simple training syllogisms and then solved 16 syllogisms in random order (see Appendix A for the complete list of syllogisms). Half of them are related to the ecological garden, half of them are related to the genetic garden; half of them are relatively simple (one mental model); half of them are relatively complex (three mental models); half of them are valid (the conclusions follow logically from premises), half of them are invalid (the conclusions do not follow logically from premises).

Each syllogism was presented in the same way. The participants were told they were to study two premises. Participants were asked to memorize the content of them. They could study each premise at their own pace, initiating the first premise and moving from the first premise to the second premise (the first premise disappeared) by pressing the space bar. Then the conclusion was presented (the second premise disappeared) and participants selected either the right arrow key (if a conclusion followed from the premises) or the left arrow key (if a conclusion did not follow from the premises). After the evaluation of the syllogism, a screen with the label “next syllogism” was presented, and when the space bar was pressed the first premise of the next syllogism was shown.

### Procedure

All participants gave informed consent for participation in study procedures. Each participant was studied individually in 2-day sessions. The first day they filled out the BDI questionnaire to assess the level of depressed mood and performed the computerized version of the OSPAN to assess the capacity of working memory. On the next day, they solved the computerized syllogism task.

## Results

### Syllogistic Reasoning Performance

#### Establishing the Existence of Belief Bias

The first, preliminary analysis aimed at demonstrating the robust existence of belief bias when assessing syllogisms concerning normal garden (believable condition) and genetic garden (unbelievable condition). For each participant, we calculated the proportion of logically correct responses in 16 syllogisms. A 2 × 2 ANOVA within-subject ANOVA [Validity (valid syllogisms, invalid syllogisms; within-subject variable)] × [Believability (believable syllogisms—normal garden, unbelievable syllogisms—genetic garden; within-subject variable)] on the proportion of correct responses yielded a main effect and an interaction effect. There was a main effect of Believability, *F*(1, 114) = 11.07, *MSE* = 0.065, *p* = 0.001, *η_*p*_^2^* = 0.089, with higher accuracy for believable syllogisms (normal garden, *M* = 0.70) compared to unbelievable syllogisms (genetic garden, *M* = 0.63).

**FIGURE 1:**
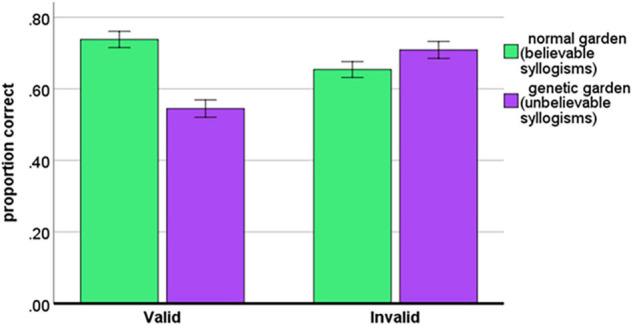
Accuracy of syllogistic reasoning as the function of Validity and Believability. Error bars represent the standard errors of the mean.

This main effect was qualified by a highly significant Validity × Believability interaction, *F*(1, 114) = 26.59; *MSE* = 0.065, *p* < 0.001, *η_*p*_^2^* = 0.189. As shown in Figure 1, the accuracy for the believable and unbelievable syllogisms was different among valid and invalid syllogisms. For believable syllogisms (normal garden), simple effect showed significantly higher accuracy for the valid than for the invalid syllogisms *t*(114) = 3.00, *p* = 0.003. In contrast (see Figure 1), for unbelievable syllogisms (genetic garden), simple effect showed a significantly lower accuracy for the valid than for the invalid syllogisms *t*(114) = 4.45, *p* < 0.001.

Because the belief bias for believable syllogisms (normal garden) and unbelievable syllogisms was reliable and similar, we applied the two basic forms of syllogisms: no-conflict syllogisms (mean correct proportions of the valid/believable and the invalid/unbelievable syllogisms) and conflict syllogisms (mean correct proportions of the valid/unbelievable and invalid/believable syllogisms) in the further analyses^4^. This decision is also consistent with the results of preliminary correlational analyses (see Table 2) between depressed mood (both continuous and dichotomized BDI scores), working memory capacity (both continuous and dichotomized OSPAN task), and syllogistic reasoning. These calculations indicate significant correlations (*p* < 0.01) only between depressed mood and conflict M1 syllogisms. Such correlations were much stronger between working memory capacity and syllogistic reasoning for conflict syllogisms M1 and M3 (although they were stronger for M3). Working memory was also strongly correlated with M1 no-conflict syllogisms but not with M3 no-conflict syllogisms (the exception was weak but a significant correlation was found between M3 no-conflict syllogisms—believable valid—for a continuous measure of OSPAN). Except for this last correlation, all the other correlations showed nearly the same results for either continuous or dichotomized measures of BDI and OSPAN (although correlations were slightly weaker for the dichotomized measures).

**TABLE 2:** Correlations between depression measure BDI (raw score and dichotomized), operation span of working memory OSPAN (raw score and dichotomized), and accuracy of syllogistic reasoning for different kinds of syllogisms.

	Normal garden				Genetic garden			
	No-conflict		Conflict		No-conflict		Conflict	
	Model 1	Model 3	Model 1	Model 3	Model 1	Model 3	Model 1	Model 3‘
BDI raw score	–0.07	0.01	−0.23*	–0.06	0.07	0.00	−0.20*	–0.08
BDI dichotomized	–0.08	0.02	−0.19*	–0.04	0.10	–0.05	−0.21*	0.01
OSPAN raw score	0.30**	0.21*	0.35**	0.55**	0.29**	–0.02	0.38**	0.51**
OSPAN dichotomized	0.20*	0.13	0.31**	0.51**	0.25**	–0.08	0.35**	0.45**

N = 115. Syllogisms differ as the function of believability (Normal Garden—believable vs. Genetic Garden—unbelievable), conflict (No-conflict vs. Conflict Syllogisms) and number of mental models (1 vs. 3). The significance levels were: *p < 0.05; **p < 0.01.

#### Reasoning Performance for No-Conflict and Conflict Syllogisms as the Function of Depressed Mood, OSPAN, and Model

To examine the role of depressed mood and operation span of working memory we used categorical variables (a low and high level of depressed mood or a low and high level of OSPAN, based on median values) instead of continuous variables in order to facilitate the interpretation of complex interaction effects. Importantly, the BDI and OSPAN scores were not correlated, Pearson’s *r* = −0.03, *p* = 0.78, and the groups created by median splits had a similar number of participants: low level of depressed mood and low level of OSPAN (*n* = 32), low level of depressed mood and high level of OSPAN (*n* = 25), high level of depressed mood and low level of OSPAN (*n* = 27), and high level of depressed mood and high level of OSPAN (*n* = 31).

A 2 × 2 × 2 × 2 mixed ANOVA [Depressed mood (low level of depressed mood, high level of depressed mood; between subject variable)] × [OSPAN (low level of OSPAN, high level of OSPAN; between subject variable)] × [Conflict (conflict syllogisms, no-conflict syllogisms; within-subject variable)] × [Model (M1 syllogisms, M3 syllogisms; within-subject variable)] on proportion of correct responses yielded four main effects and three interaction effects with depressed mood. We also inspected two additional strong interaction effects involving the Model variable.

There was a main effect of Depressed mood, *F*(1, 111) = 4.09, *MSE* = 0.088, *p* = 0.046, *η_*p*_^2^* = 0.036, with a higher accuracy among participants with a low level of depressed mood (*M* = 0.69) compared to participants with a high level of depressed mood (*M* = 0.63). There was also a very strong main effect of OSPAN, *F*(1, 111) = 51.35, *MSE* = 0.088, *p* < 0.001, *η_*p*_^2^* = 0.316, with a much higher accuracy among participants with a high level of OSPAN (*M* = 0.76) compared to participants with a low level of OSPAN (*M* = 0.56). Moreover, there was a strong main effect of Conflict *F*(1, 111) = 35.32, *MSE* = 0.050, *p* < 0.001, *η_*p*_^2^* = 0.241, with a much higher accuracy for no-conflict syllogisms (*M* = 0.72) compared to conflict syllogisms (*M* = 0.60). Finally, there was also a strong main effect of Model *F*(1, 111) = 14.98, *MSE* = 0.043, *p* < 0.001, *η_*p*_^2^* = 0.119, with a higher accuracy for simpler M1 syllogisms (*M* = 0.70) compared to more complex M3 syllogisms (*M* = 0.62).

**FIGURE 2:**
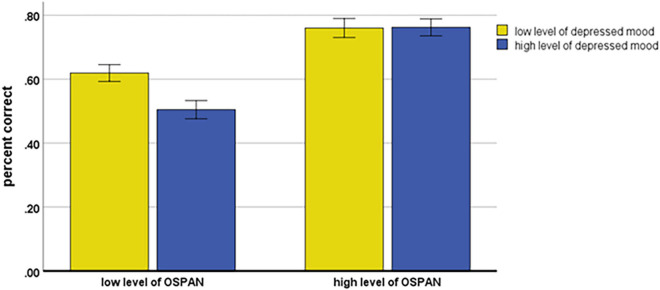
Accuracy of syllogistic reasoning as the function of Depressed mood and OSPAN. Error bars represent the standard errors of the mean.

The main effects of Depressed mood and OSPAN were qualified by a significant Depressed mood × OSPAN interaction, *F*(1, 111) = 4.40; *MSE* = 0.088, *p* = 0.038, *η_*p*_^2^* = 0.038. As shown in Figure 2, accuracy among the low and high levels of depressed mood participants was different among those with relatively high or low levels of OSPAN. For participants with a relatively low level of OSPAN, a simple effect showed a significantly higher accuracy for low than for high level of depressed mood participants *t*(114) = 2.95, *p* = 0.004. In contrast (see Figure 2), for participants with a relatively high level of OSPAN, simple effect showed a very similar and relatively high accuracy of syllogistic reasoning in both low and high levels of depressed mood participants (*t* < 1).

**FIGURE 3:**
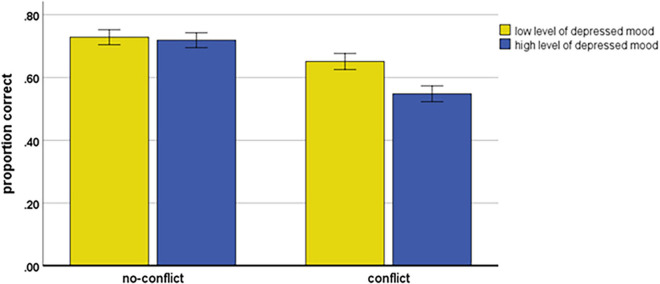
Accuracy of syllogistic reasoning as the function of Depressed mood and Conflict. Error bars represent the standard errors of the mean.

The main effects of Depression and Conflict were qualified by a significant Depressed mood × Conflict interaction, *F*(1, 111) = 5.02; *MSE* = 0.05, *p* = 0.027, *η_*p*_^2^* = 0.043. As shown in Figure 3, accuracy among the participants with low and high levels of depressed mood was different among conflict and no-conflict syllogisms. For conflict syllogisms, simple effect showed significantly higher accuracy for low than for high level of depressed mood participants *t*(111) = 2.87, *p* = 0.005. In contrast (see Figure 3), for no-conflict syllogisms, simple effect showed a very similar and relatively high accuracy of syllogistic reasoning in both participants with low and high levels of depressed mood (*t* < 1).

**FIGURE 4:**
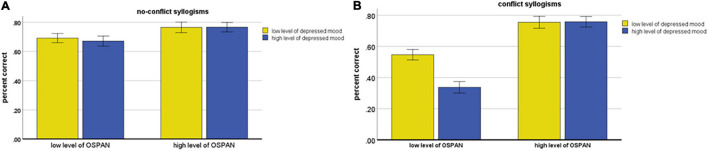
Accuracy of syllogistic reasoning as the function of Depressed mood and OSPAN for no-conflict syllogisms **(A)** and conflict syllogism tables **(B)**. Error bars represent the standard errors of the mean.

The above discussed interactions were additionally qualified by a significant Depressed mood × OSPAN × Conflict interaction, *F*(1, 111) = 5.22; *MSE* = 0.050, *p* = 0.024, *η_*p*_^2^* = 0.045. To disentangle this complex interaction we assumed that the category of syllogisms—conflict or no-conflict—was a moderator variable and conducted mixed ANOVAs separately for the no-conflict and conflict syllogisms. For the no-conflict syllogisms this analysis showed only the main effect of OSPAN *F*(1, 111) = 6.28; *MSE* = 0.064, *p* = 0.014, *η_*p*_^2^* = 0.054 with higher accuracy among a high level of OSPAN than a low level of OSPAN in participants (see Figure 4A).

For the conflict syllogisms this analysis showed the main effect of depressed mood *F*(1, 111) = 8.24; *MSE* = 0.073, *p* = 0.005, *η_*p*_^2^* = 0.069 and the main effect of OSPAN *F*(1, 111) = 76.74; *MSE* = 0.073, *p* < 0.001, *η_*p*_^2^* = 0.409; however, these main effects were qualified by a significant Depressed mood × OSPAN interaction, *F*(1, 111) = 8.74; *MSE* = 0.073, *p* = 0.004, *η_*p*_^2^* = 0.073. As shown in Figure 4B, accuracy for low and high level of depressed mood participants was different between low and high level of OSPAN participants. Among the low OSPAN participants, simple effect showed a significantly higher accuracy in low than high level of depressed mood participants *t*(111) = 4.00, *p* < 0.001. In contrast (see Figure 4B), for participants with a relatively high level of OSPAN, simple effect showed a very similar and relatively high accuracy of syllogistic reasoning in both low and high level of depressed mood participants (*t* < 1)^5^.

The four-way mixed ANOVA also revealed the existence of three additional interaction effects, involving Model (complexity of syllogisms) and OSPAN; however, these interactions did not concern the depressed mood variable. Because they are not relevant to the major issue of this paper, we only indicate the most important simple effects of those interactions. Interaction Conflict × OSPAN [*F*(1, 111) = 30.37; *MSE* = 0.050, *p* < 0.001, *η_*p*_^2^* = 0.215] showed that significant differences between low and high level of OSPAN participants existed for the conflict syllogisms only. Interaction Conflict × Model [*F*(1, 111) = 13.47; *MSE* = 0.042, *p* < 0.001, *η_*p*_^2^* = 0.108] revealed that significant differences between conflict and no-conflict syllogisms were visible only for more complex M3 syllogisms. Finally, the interaction Conflict × OSPAN × Model [*F*(1, 111) = 11.69, *MSE* = 0.042, *p* = 0.001, *η_*p*_^2^* = 0.095] showed the most significant differences between participants with low and high levels of OSPAN when considering conflict M3 syllogisms.

#### Processing Time

A 2 × 2 × 2 × 3 mixed ANOVA (Depressed mood [low level of depressed mood, high level of depressed mood; between subject variable)] × [OSPAN (low level of OSPAN, high level of OSPAN; between subject variable)] × [Model (M1 syllogisms, M3 syllogisms; within-subject variable)] × [Phase (first premise, second premise, conclusion)] on processing time yielded two main effects and one interaction effect. There were no main effects of depressed mood and OSPAN and no interaction effects of those variables with other variables.

There was a very strong main effect of Phase *F*(2, 222) = 209.05, *MSE* = 2.09, *p* < 0.001, *η_*p*_^2^* = 0.653, with much longer time spent on conclusions (*M* = 8.31 s) compared to time spent on a second premise (*M* = 6.35 s) and time spent on a first premise (*M* = 5.40 s), all differences *p* < 0.001 (*post hoc* Sidak test was applied). There was also a strong main effect of Model *F*(1, 111) = 25.74, *MSE* = 1.83, *p* < 0.001, *η_*p*_^2^* = 0.188, with shorter time spent on M1 syllogisms (*M* = 6.36 s) compared to time spent on more complex M3 syllogisms (*M* = 6.89 s).

For the significant Model × Phase interaction, *F*(2, 222) = 8.75; *MSE* = 1.63, *p* < 0.001, *η_*p*_^2^* = 0.073, simple effects showed that there were no reliable differences for M1 and M3 syllogisms in studying the first premise (*t* = 1.10, *p* = 0.28); however, the second premise (*t* = 2.12, *p* = 0.036) and especially the conclusion (*t* = 5.07, *p* < 0.001) were analyzed much longer for M3 than for M1 syllogisms.

An additional 2 × 2 × 2 mixed ANOVA examining the role of distinction on conflict and no-conflict syllogisms [Depressed mood (low level of depressed mood, high level of depressed mood; between subject variable)] × [OSPAN (low level of OSPAN, high level of OSPAN; between subject variable)] × [Conflict (conflict syllogisms, no-conflict syllogisms; within-subject variable)] did not yield any main effects nor interaction effects for the processing time^6^.

### General Discussion

Gathered research findings provide further compelling evidence for the cognitive exhaustion model of subclinical depression (von Hecker and Sedek, 1999; Sedek and von Hecker, 2004; von Hecker et al., 2013) that depressed mood substantially impairs generative mental models activity; in the current study specifically related to building correct mental models of syllogistic reasoning. There was the significant main effect of the level of depressed mood on limitations in syllogistic reasoning, however, in line with predictions, this relationship was strongly moderated by individual differences in working memory capacity and by the construction of syllogisms (i.e., whether or not participants solved conflict or no-conflict tasks).

The highly significant main effect of OSPAN on deductive reasoning accuracy is consistent with the replicated findings demonstrating moderate or strong correlations between OSPAN and syllogistic reasoning on student samples (Copeland and Radvansky, 2004; De Neys et al., 2005; Robison and Unsworth, 2017). There are two main interpretations of shared variance of working memory capacity and syllogistic reasoning. The first argument is that participants with higher working memory have resources to develop relevant counter examples. The second argument is that participants with higher working memory are able to develop more than one mental model.

In the following sections we discuss first the findings concerning reaction times related to the issue of potential differences of general motivation. Next, we summarize the findings related to the moderating role of operating span of working memory (OSPAN) and finally we extend and integrate them with the issue of inhibition (i.e., belief bias) inherent for the construction of conflict syllogisms.

Reaction times for participants with low and high levels of depressed mood showed a very similar pattern of results. Both groups of students, with high and low depressed mood, spent more time analyzing the second premise (enabling construction of a tentative mental model) than the first premise and they devoted the longest time for formulating the conclusion (whether the conclusion follows or does not follow from the premises). Both participants with high and low depressed mood spent more time analyzing the second premise and deciding among conclusions when solving more difficult M3 syllogisms than solving simpler M1 syllogisms. Thus, the pattern of processing time helps to refute an explanation of the reasoning deficit in terms of a generally lowered motivation to solve syllogism tasks in participants with a high level of depressed mood. These findings replicated previous results on very similar patterns of study times among nondepressed and depressed participants for other reasoning tasks based on generative mental model building, such as constructing social cliques (von Hecker and Sedek, 1999) or solving linear syllogisms (Sedek and von Hecker, 2004).

An important finding of our study showed that depressed mood (measured by the BDI) is not correlated with OSPAN. This finding, replicating the previous research on depression and linear syllogisms (Sedek and von Hecker, 2004), refutes the hypothesis that working memory capacity mediates the relationship between depressed mood and limitations in solving categorical or linear syllogisms. However, the findings confirmed the predicted role of working memory as the moderator of depressed mood effects on impaired syllogistic reasoning. Namely, participants with a relatively high level of operation span (both high and low in levels of depressed mood) solved the categorical syllogisms similarly and efficiently (appr. 80% reasoning accuracy). However, among the participants with a relatively low level of working memory capacity the reasoning accuracy was significantly lower among participants with high compared to low level of depressed mood (see Figure 2).

Our results seem to be also incompatible with the capacity limitation view of depression (Ellis and Ashbrook, 1988; Ellis et al., 1997). This explanation suggests that cognitive capacity is reduced in depression because depressed people allocate a portion of their attentional resources to ruminative thoughts or task-irrelevant features of the task. This capacity limitation model suggests we should have observed a disproportional decrease in performance as a function of mental model difficulty in the group with a high level of depressed mood, as compared to the group with a low level of depressed mood. In fact however, our findings did not show such interaction: all participants (both with high and low levels of depressed mood) showed worse performance for more difficult M3 syllogisms than for M1 syllogisms. These findings for categorical syllogisms and previous findings for linear syllogisms (Sedek and von Hecker, 2004) indicate the genuine integration deficit among participants with depressed mood and confirm predictions of the cognitive exhaustion model (Sedek and Kofta, 1990; Kofta and Sedek, 1998; von Hecker and Sedek, 1999; von Hecker et al., 2013).

The next issue of these main findings is related to the examination of the prediction that the content effects causing belief bias in reasoning might especially affect participants with a high level of depressed mood. We had objections for using the unbelievable syllogisms that contain obviously absurd premises (like “all parents are mothers”). We assumed that especially participants with a high level of depressed mood might be demotivated and limit their involvement in effortful thinking when they are forced to solve syllogisms which are completely inconsistent with reality. Therefore, in this study we constructed a unique and original set of syllogisms. Interestingly, on the domain of conditional reasoning, researchers (Markovits et al., 2017) also designed a conceptually similar scenario based on the rules observed on a fictitious planet called Kronus.

Confirming our assumptions, the scenario with two kinds of gardens prompted the emergence of a very strong belief bias (see Figure 1). For the whole sample, we yielded very similar belief bias for the normal garden (believable condition; accuracy for valid syllogisms was higher than for invalid syllogisms) as for the genetic garden (unbelievable condition; accuracy for invalid syllogisms was higher than for valid syllogisms). Therefore, in further analyses we compared two kinds of conflict syllogisms (containing the conflict between conclusions based on beliefs and conclusions based on logical reasoning) and no-conflict syllogisms (not containing the conflict between beliefs and logical reasoning).

The results confirmed the general prediction that belief-based reasoning often prompts incorrect responses among participants with a high level of depressed mood that conflict with the logically appropriate response. The series of consecutive interaction effects refined the results and showed the moderating roles of both construction of syllogism (i.e., conflict versus no-conflict) and individual differences in working memory. Significant interaction Depressed mood × Conflict × OSPAN showed that limitations related to a high level of depressed mood disappeared for the no-conflict syllogisms (see Figure 4A). However, in the case of the conflict syllogisms, it was clear that a high level of OSPAN works as a “cognitive shield” (see Figure 4B): both participants with high and low levels of depressed mood, and characterized by high working memory capacity, solved conflict syllogism tasks quite well. However, in the case of a low level of working memory capacity, the performance in conflict syllogism tasks was significantly lower in participants with a high level of depressed mood than in those with a low level of depressed mood.

This issue of solving conflict versus no-conflict syllogisms has been analyzed in detail by dual-process theoretical concepts (e.g., Stanovich and West, 2000; Evans, 2003, 2006; Handley et al., 2004; De Neys, 2006; Markovits et al., 2017). According to these concepts, logically correct syllogistic reasoning in the case of belief-logic conflict demands that participants temporarily inhibit their beliefs and refrain from taking them into account. Furthermore, these concepts assume that in the case of conflict syllogisms reasoners will engage in the analytical system and resist heuristic-based responses. Additionally, it is assumed that this analytical system is strongly related to the cognitive capacity of working memory. That is, individuals with greater working memory capacity are more likely to inhibit the heuristic cued response. Why individual differences in working memory span measures like OSPAN might be strongly related with solving conflict syllogisms? According to Engle and Kane (2004), there are two important factors that explain why OSPAN is a strong predictor of complex attentional and reasoning tasks: goal-maintenance and conflict resolution. As indicated by the series of studies of Randall Engle and his associates (Kane and Engle, 2003; Redick et al., 2011; Shipstead and Engle, 2013), participants with a relatively low level of OSPAN in comparison to participants with a relatively high level of OSPAN had substantially lower performance in cognitive tasks that demand inhibition of prepotent response like the Stroop task, anti-saccade task, or go/no-go task. Therefore, we expected and confirmed the generally effective inhibition of prepotent responses in conflict syllogisms among participants relatively high in OSPAN. Participants’ problem in conflict syllogisms is that belief-based reasoning often prompts incorrect responses that interfere with the logically appropriate response. The results showed that this interference was successfully overpassed by both participants with low and high levels of depressed mood given a relatively high level of OSPAN. However, in the case of a low level of OSPAN, participants with a high level of depressed mood were less able than participants with a low level of depressed mood to resolve such interference in the case of conflict syllogisms.

It is important to note a potential alternative account for the present findings. Recent research in the dual process field have shown that good reasoners do not always need to deliberate to correct erroneous intuitive responses. Often they will generate the correct answer intuitively (e.g., Bago and De Neys, 2017). Moreover, this tendency has been linked to cognitive capacity (e.g., Raoelison et al., 2020). According to the presented research evidence, depressive mood interferes with deliberate reasoning. According to the first theoretical account, reasoning performance of depressed participants with a higher span of working memory might suffer less because their higher resources allow them to still deliberate better (e.g., deliberately inhibiting erroneous belief-based responses) than depressed participants with a lower span of working memory. However, according to the alternative account, based on the evidence cited above, it might also be that participants with high spans of working memory simply intuit better. Hence, if depressive mood primarily affects deliberate reasoning, the soundly intuiting participants with a higher span of working memory will not be affected. Not because they are better deliberators per se but rather because they are better intuitors. To conclude this alternative account, depressed participants with a high span of working memory might sidestep the negative impact of depressive mood on deliberate thinking because sound reasoning requires less deliberation for them from the outset. These two accounts are not mutually exclusive and it would be a good idea to examine and contrast predictions of them in further research.

It is important to note again that the impairment related to a high level of depressed mood in solving conflict syllogisms was not related to the complexity level of syllogisms (there was a lack of any interaction of Depressed mood × Model), hence it consistently confirmed the predictions of the cognitive exhaustion model (von Hecker and Sedek, 1999; Sedek and von Hecker, 2004; von Hecker et al., 2013). However, the relationship between working memory capacity and solving conflict syllogisms was stronger for more difficult M3 models than for more simple M1 models. This distinctive pattern of relationships between depressed mood, working memory capacity, and syllogistic reasoning indicates that high working memory capacity is a buffering variable and acts as a protective factor preventing the negative impact of depressed mood on syllogistic reasoning.

#### Limitations and Future Research

The obvious limitation is that this study is correlational in design and no causal explanations are possible between depressed mood and syllogistic reasoning. Future research with an intensive longitudinal design is recommended.

The next limitation is that the gathered evidence indicating the role of moderating variables on syllogistic reasoning is based on a single study and extended replications are needed to establish the robustness of the findings. The critical interaction effect of Depressed mood × Conflict × OSPAN is based on a median split of the working memory and BDI scores. This implies that there are only about 30 participants in each of the critical WM (working memory) /BDI (depressed mood) groups. Hence, there still exists substantial uncertainty of how robust the results really are. The most promising form of extended replication would be an experimental test of the main hypothesis. For example, a dual task design could be used (such as the popular dot memorization task: Miyake et al., 2001) and burden participant’s working memory with a secondary task while they were solving the syllogisms tasks (see De Neys and Schaeken, 2007; Franssens and De Neys, 2009; Bago and De Neys, 2017 for research with such a secondary load task in the reasoning problems). If the proposed hypothesis is right one would expect that depressive mood will have a stronger negative impact under these conditions. Especially, depressed participants with a relatively high span of working memory under load should show similarly worsened syllogistic reasoning as depressed participants with a relatively low span of working memory.

The specific limitation of the current sample is the very restricted age of participants (18–19 years old). Myelination of white matter connected with working memory, as measured by fractional anisotropy (FA), progresses nonlinearly with age and for most of the tracts stabilizes at the age of 20 (Chang et al., 2015; Lazar, 2017; Figure 4). However, some tracts reach maturation in the later period, i.e., 23 years for the corticospinal tract, 27 years for the cingulum, and even more than 30 years for the uncinate fasciculus (Lebel et al., 2019; Figure 4). Taking that into account, it is a question whether our main results (interaction between depressed mood and working memory) would also hold across a broader age range of population. In order to determine that, it would be required to conduct research on a larger and more representative sample. As indicated by a recent meta-analysis of predictors of reasoning across adult life-span based on 119 studies (Verhaeghen, 2011; Figure 3, p. 179), both processing speed and working memory (OSPAN was applied there as one of the most popular measures of working memory capacity) were strong sequential mediators of the relationship between age and complex cognition (defined mostly by reasoning tests). Therefore, the further broader age range research should include measures of both working memory and processing speed. The directly relevant study of the age differences in solving conflict and no-conflict syllogisms (De Neys and Van Gelder, 2009) showed no age differences in solving no-conflict syllogisms but strong age differences in solving conflict syllogisms (the performance of younger adults was better compared to older adults and younger children). However, age differences were not mediated by the composite measure of capacity to resist intuitive thinking in decision making tasks. In the extended studies it would be important to include not only the measures of evaluated validity of conclusions but also measures of confidence judgments. There is an ongoing debate about the mechanisms that generate different accuracy effects for conflict and no-conflict syllogisms (i.e., related to the belief-bias phenomenon) between an approach based on mental models (e.g., Oakhill et al., 1989; Garnham and Oakhill, 2005) and more recent approaches based on signal detection frameworks such as argument strength explanation (e.g., Klauer and Kellen, 2011) or response bias account (e.g., Dube et al., 2010; Trippas et al., 2013, 2018; Stephens et al., 2019). The examinations of the predictions of those competing theoretical models for explaining limitations related to a high level of depressed mood in solving conflict syllogisms demand an examination of both validity and confidence judgements.

The important issue of further research would be manipulating the emotional contents of materials (neutral versus emotionally related to concerns of participants with depressed mood). As indicated in the introduction, recent research demonstrated that in conditions where emotional contents of reasoning tasks are personally relevant, logical reasoning often improves. It would be especially interesting to analyze, using the advanced research scheme (c.f., Blanchette et al., 2014), whether or not emotional content among participants with a high level of depressed mood would eliminate the limitations in solving conflicting syllogisms and whether it would be again moderated by working memory capacity.

Stanovich and West (2000) have shown that thinking disposition also explains the unique variance of belief-independent reasoning independent of working memory capacity. They refer the term thinking dispositions to a range of personality characteristics that include openness to ideas, faith in intuition, and cognitive flexibility. An interesting future line of research would be to examine the extent to which limitations related to a high level of depressed mood in syllogistic reasoning is related to lowered levels of those positive thinking dispositions.

Finally, the existing research showed the important similarities between theoretical explanations of categorical syllogisms and conditional reasoning and the role of working memory capacity (e.g., Markovits et al., 2002; De Neys et al., 2005). Therefore, the natural extension of the research showing the impaired reasoning would be examination of the potential limitations among participants with a high level of depressed mood in conditional reasoning and examining whether the high level of working memory span is again the important buffering variable.

## Conclusion

These findings indicating the strong buffering role of high working memory span on the relationship between depressed mood and syllogistic reasoning in young adults have important implications for cognitive forms of therapy of depression symptoms (for review: Motter et al., 2016), such as advantages of improving working memory capacity of depressive persons with the use of specially designed cognitive trainings. Such cognitive training interventions should be specifically recommended for participants with high levels of depressed mood and with relatively low levels of working memory capacity. Essential elements of novel forms of cognitive training, aimed at eliminating impairments related to a high level of depressed mood in cognitive functioning, should also include a variety of tasks focused on skill enhancement in generating mental models.

## Data Availability Statement

The raw data supporting the conclusions of this article will be made available by the authors, without undue reservation.

## Ethics Statement

Ethical review and approval was not required for the study on human participants in accordance with the local legislation and institutional requirements. The patients/participants provided their written informed consent to participate in this study.

## Author Contributions

GS developed the study concept. JW and GS drafted the manuscript. KR provided the revisions. All authors performed the data analysis and interpretation, and approved the final version of the manuscript for submission.

## Conflict of Interest

The authors declare that the research was conducted in the absence of any commercial or financial relationships that could be construed as a potential conflict of interest.

## Publisher’s Note

All claims expressed in this article are solely those of the authors and do not necessarily represent those of their affiliated organizations, or those of the publisher, the editors and the reviewers. Any product that may be evaluated in this article, or claim that may be made by its manufacturer, is not guaranteed or endorsed by the publisher.

## Footnotes

1 We use the term *depressed mood* to denote that the assignment of participants in the groups with relatively high or relatively low levels of depressed mood was made on the basis of the BDI scores (Beck, 1967). We avoided using the term *sad mood* because it is often applied to refer to mood manipulation procedures, which are not used in the presented research nor in the reported research program.

2 Channon and Baker (1994) did not recruit participants with BDI scores between 5 and 10, thus there are larger differences in the nondepressed group in comparison with this study.

3 The mean of the OSPAN measure in this sample (*M* = 33.51) was similar to the mean of a recent study on the same topic on a student sample (more than 1 year older), *M* = 36.85 (Robison and Unsworth, 2017).

4 Such classification also helps to avoid puzzling interpretation of the significant four-way interaction effect: Validity × Believability × Depressed mood × OSPAN.

5 We confirmed these most important findings, namely the significant interaction of Depressed mood × OSPAN for conflict syllogisms, also when applying continuous variables instead of categorical variables. We used SPSS version of the Process macro 3.5 (Hayes, 2018). For *n* = 10,000 bootstrap resamples, 95% confidence intervals, we revealed the significant interaction effect of continuous variables Depressed mood and OSPAN for conflict syllogisms, *t*(111) = 2.41, *p* = 0.017.

6 Adding the Conflict variable to the previous mixed ANOVA design yielded a significant, however, difficult for interpretation, interaction effect: Model × Phase × Depressed mood × Conflict.

## Appendix

**TABLE A1:** List of syllogisms in the computerized test.

Syllogism	Proportion correct	Pattern	Model	Garden	Logic
All grapes are ripe. Everything ripe glows in the dark. All grapes glow in the dark.	0.55	All A are B All B are C All A are C	1M	G	T
All fruits are batteries. All apples are fruits. All apples are batteries.	0.63	All B are A All C are B All C are A	1M	G	T
All ants have wings. Everything winged sings. Everything that sings is an ant.	0.69	All A are B All B are C All C are A	1M	G	F
All parrots have horns. All red birds are parrots. Everything with horns is a red bird.	0.70	All B are A All C are B All A are C	1M	G	F
All grasses are green. Everything green is alive. All grasses are alive.	0.74	All A are B All B are C All A are C	1M	N	T
All plums have seeds. All Mirabelle prunes are plums. All Mirabelle prunes have seeds.	0.78	All B are A All C are B All C are A	1M	N	T
All the wormy fruits fall from the tree. All the fruits that fall from the tree are overripe. All overripe fruits are wormy.	0.77	All A are B All B are C All C are A	1M	N	F
Everything healthy is sour. Everything yellow is healthy. Everything sour is yellow.	0.75	All B are A All C are B All A are C	1M	N	F
No edible product is a toadstool. Some jumping mushrooms are toadstools. Some jumping mushrooms are not edible.	0.44	No A are B Some C are B Some C are not A	3M	G	T
All bilberries speak Polish. No blueberries are bilberries. Some fruits that speak Polish are not blueberries.	0.57	All A are B No C are A Some B are not C	3M	G	T
No smiling apples grow on the bushes. Some pretty fruits grow on bushes. Some smiling apples are not pretty fruits.	0.69	No A are B Some C are B Some A are not C	3M	G	F
All tigers have two tails. No horse is a tiger. Some horses do not have two tails.	0.76	All A are B No C are A Some C are not B	3M	G	F
No dogs have colorful feathers. Some ducks have colorful feathers. Some ducks are not dogs.	0.63	No A are B Some C are B Some C are not A	3M	N	T
All moths fly at night. No bats are moths. Some animals that fly at night are not bats.	0.80	All A are B No C are A Some B are not C	3M	N	T
No hare is a fox. Some devious animals are foxes. Some hares are not devious.	0.56	No A are B Some C are B Some A are not C	3M	N	F
Everything that runs breathes. No frog is running. Some frogs do not breathe.	0.54	All A are B No C is A Some C are not B	3M	N	F

*Syllogisms—the first premise, the second premise, conclusion. Proportion correct—proportion of correctly solved syllogisms for all participants. Pattern—a general pattern of the construction of a syllogism, abstracted from the content. Model—syllogism difficulty level: 1M (easy), 3M (difficult). Garden—a type of garden to which the reality of the syllogism applies: N (natural), G (genetic). Logic—whether or not the conclusion follows logically from the premises: T (True—it follows), F (False—it does not follow).*
